# Supplementary material for: A hygroscopic nano-membrane coating achieves efficient vapor-fed photocatalytic water splitting
Source: Nat Commun. 2022 Sep 28;13:5698. doi: 10.1038/s41467-022-33439-x (PMC9519874; doi:10.1038/s41467-022-33439-x)
Supplement: Supplementary file 1 — Supplementary Information [file 41467_2022_33439_MOESM1_ESM.pdf]

A hygroscopic nano-membrane coating achieves efficient vapor-fed photocatalytic water splitting

Takuya Suguro[1], Fuminao Kishimoto[1], Nobuko Kariya[2], Tsuyoshi Fukui[2], Mamiko Nakabayashi[3], Naoya Shibata[3], Tsuyoshi Takata[4], Kazunari Domen[4,5], Kazuhiro Takanabe[1].*

[1] Department of Chemical System Engineering, School of Engineering, The University of Tokyo. 7-3-1 Hongo, Bunkyo-ku, Tokyo 113-8656, Japan.

[2] Science & Innovation Center, Mitsubishi Chemical Corporation, 1000 Kamoshida-cho, Aoba-ku, Yokohama, Kanagawa 227-8502, Japan

[3] Institute of Engineering Innovation, School of Engineering, The University of Tokyo, 7-3-1 Hongo, Bunkyo-ku, Tokyo 113−8656, Japan

[4] Research Initiative for Supra-Materials (RISM), Shinshu University, 4-17-1 Wakasato, Nagano 380−8553, Japan

[5] Office of University Professors, The University of Tokyo, 7-3-1 Hongo, Bunkyo-ku, Tokyo 113−8656, Japan

Corresponding Author

*E-mail: takanabe@chemsys.t.u-tokyo.ac.jp

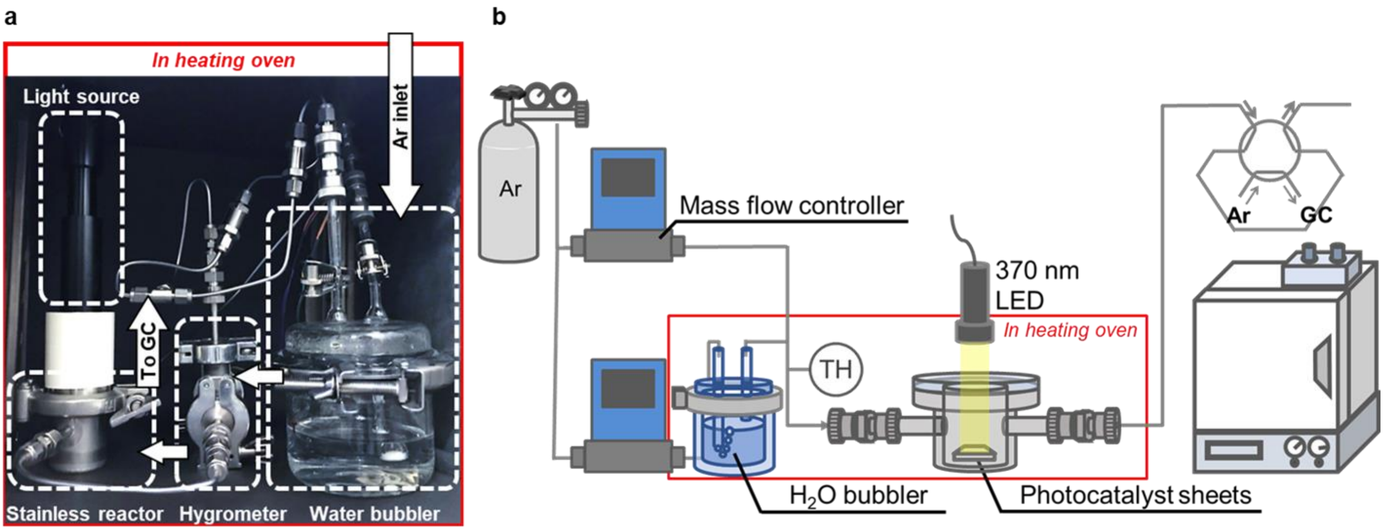

Supplementary Fig. 1: Photographs (a) and schematic (b) of a vaper feeding photocatalytic overall water splitting system. Humidity is controlled by mixing dry Ar and water vapor-saturated Ar. The reaction temperature was controlled via a heating oven (297–353 K). The reaction temperature and humidity were detected via a hygrothermograph (TH).

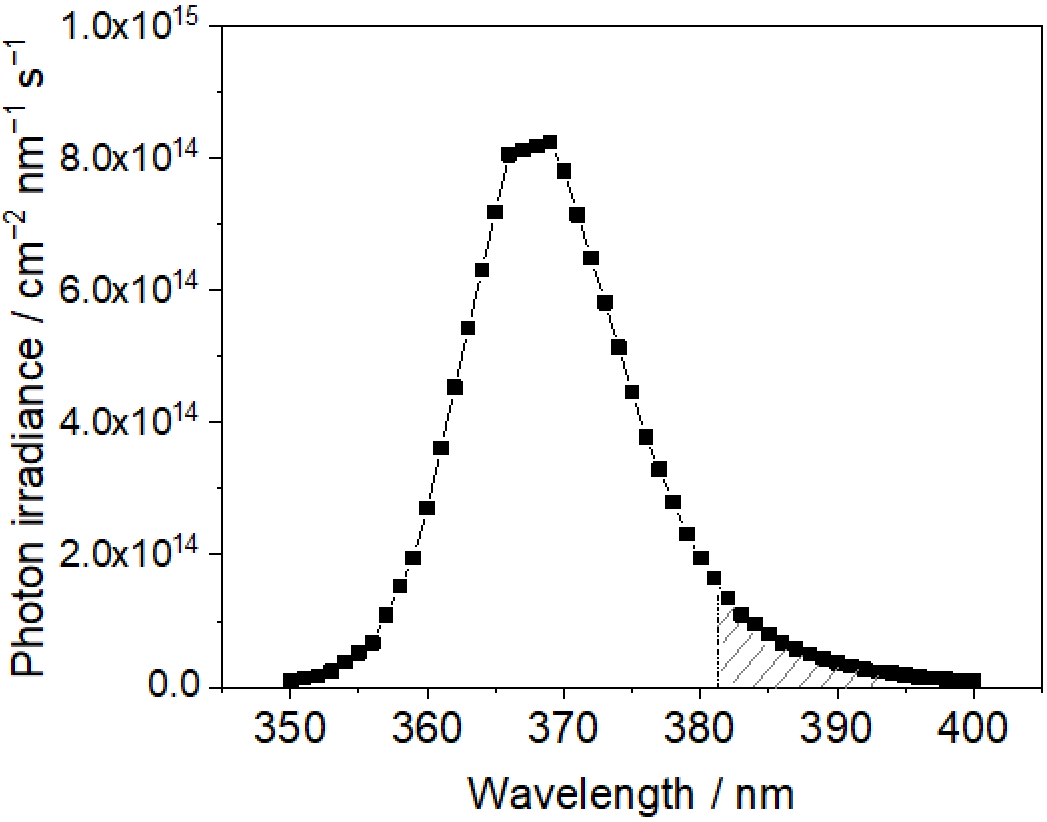

Supplementary Fig. 2: Photon irradiance distribution used for photocatalytic reaction. To calculate the photon flux, photons at wavelengths above 381 nm (which $SrTiO_3$ cannot absorb from the UV-Vis results) were excluded in the shaded area.

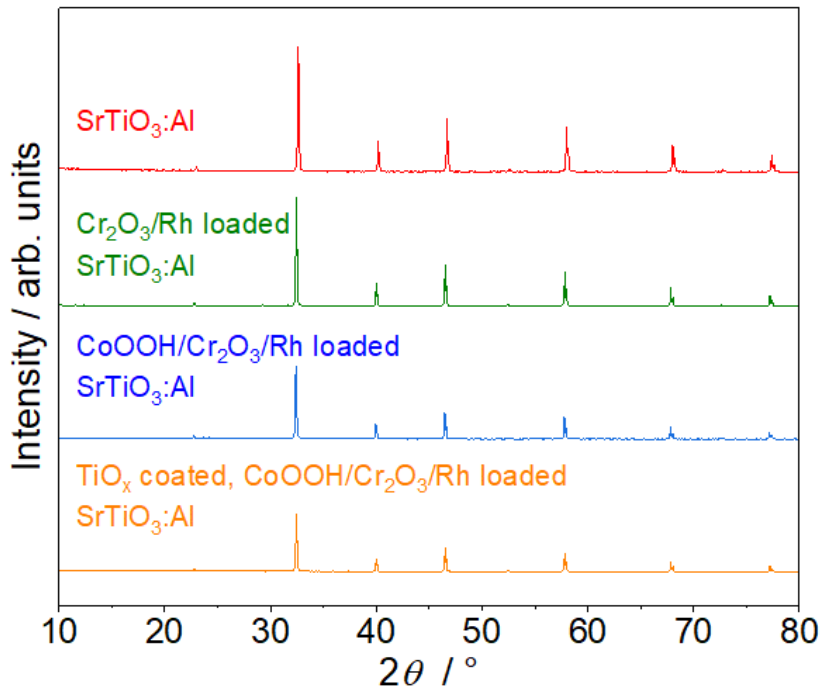

Supplementary Fig. 3: XRD patterns of the photocatalysts. The XRD patterns for pristine $SrTiO_3$:Al (without cocatalyst), $SrTiO_3$:Al with Cr_2O_3/Rh, $SrTiO_3$:Al with CoOOH and Cr_2O_3/Rh, and TiO_x coated $SrTiO_3$:Al with CoOOH and Cr_2O_3/Rh.

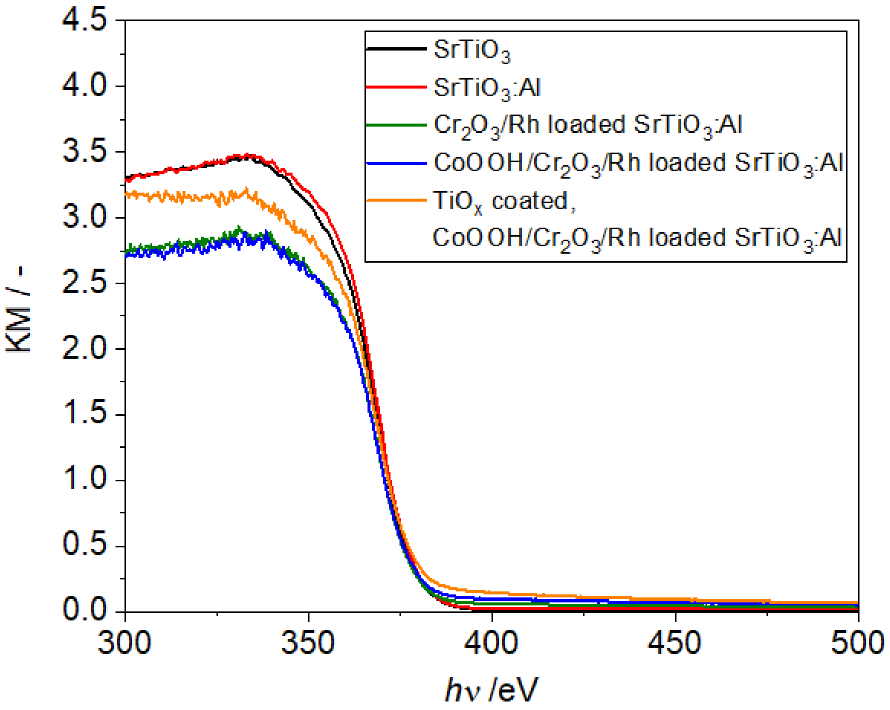

Supplementary Fig. 4: UV-Vis spectra of the photocatalysts. The diffuse reflectance spectra for pristine $SrTiO_3$:Al (without cocatalyst), $SrTiO_3$:Al with Cr_2O_3/Rh, $SrTiO_3$:Al with CoOOH and Cr_2O_3/Rh, and TiO_x coated $SrTiO_3$:Al with CoOOH and Cr_2O_3/Rh.

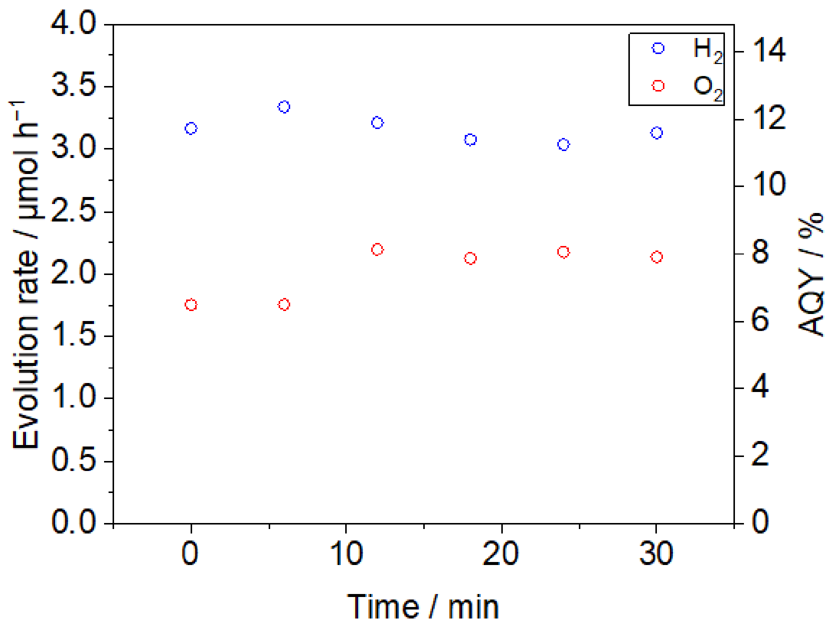

Supplementary Fig. 5: Dependence of H_2 and O_2 evolution rate of $RhCrO_x$ loaded $SrTiO_3$:Al. These demonstrations were under saturated water vapor balanced with Ar at 24 °C (10 mL min^{-1}, p_{H2O} = 2.9 kPa). Light source: 370 nm LED (5.1 mW cm^{-2}).

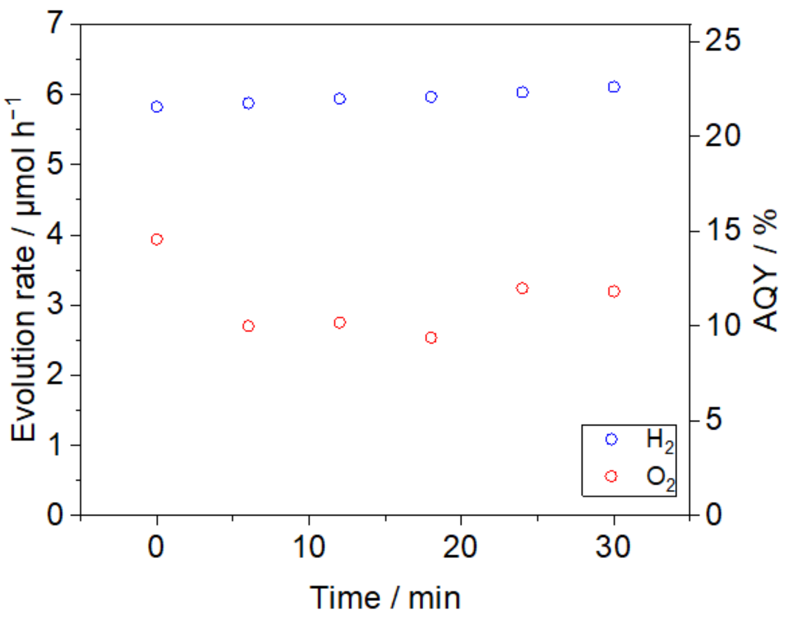

Supplementary Fig. 6: Dependence of H_2 and O_2 evolution rate of CoOOH/Rh loaded $SrTiO_3$:Al. These demonstrations were under saturated water vapor balanced with Ar at 24 °C (10 mL min^{-1}, p_{H2O} = 2.9 kPa). Light source: 370 nm LED (5.1 mW cm^{-2}).

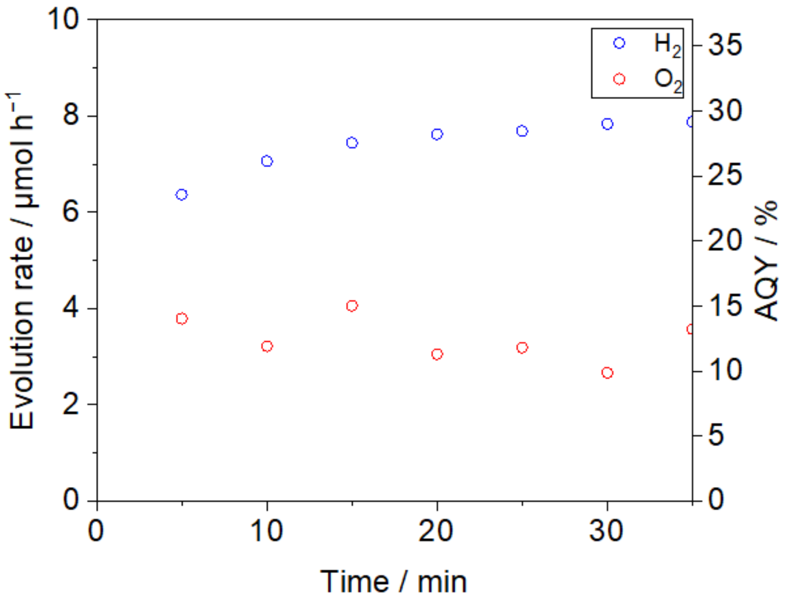

Supplementary Fig. 7: Dependence of H_2 and O_2 evolution rate of $CoOOH/Cr_2O_3/Rh$ loaded $SrTiO_3$:Al. These demonstrations were under saturated water vapor balanced with Ar at 24 °C (10 mL min^{-1}, p_{H2O} = 2.9 kPa). Light source: 370 nm LED (5.1 mW cm^{-2}).

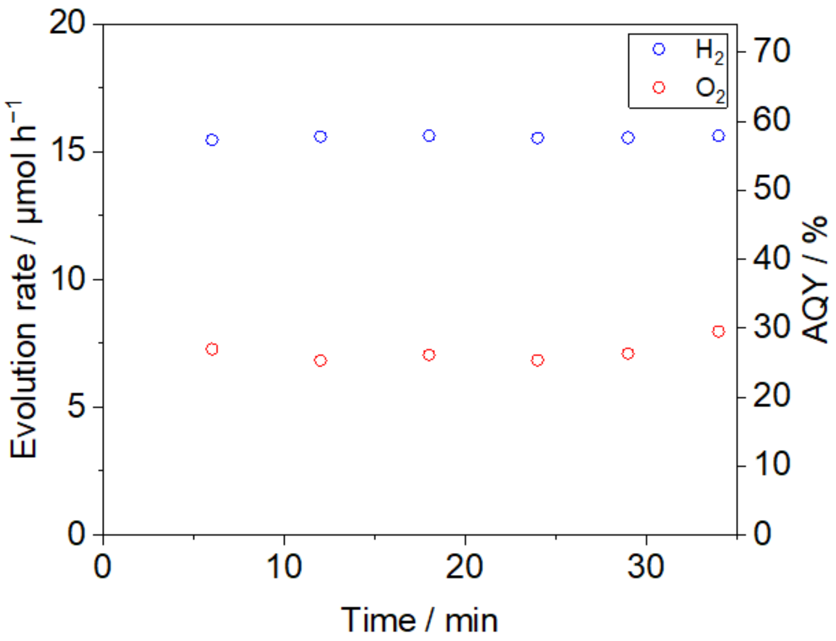

Supplementary Fig. 8: Dependence of H_2 and O_2 evolution rate of TiO_x coated, CoOOH/Rh loaded $SrTiO_3$:Al.

These demonstrations were under saturated water vapor balanced with Ar at 24 °C (10 mL min^{-1}, p_{H2O} = 2.9 kPa). Light source: 370 nm LED (5.1 mW cm^{-2}).

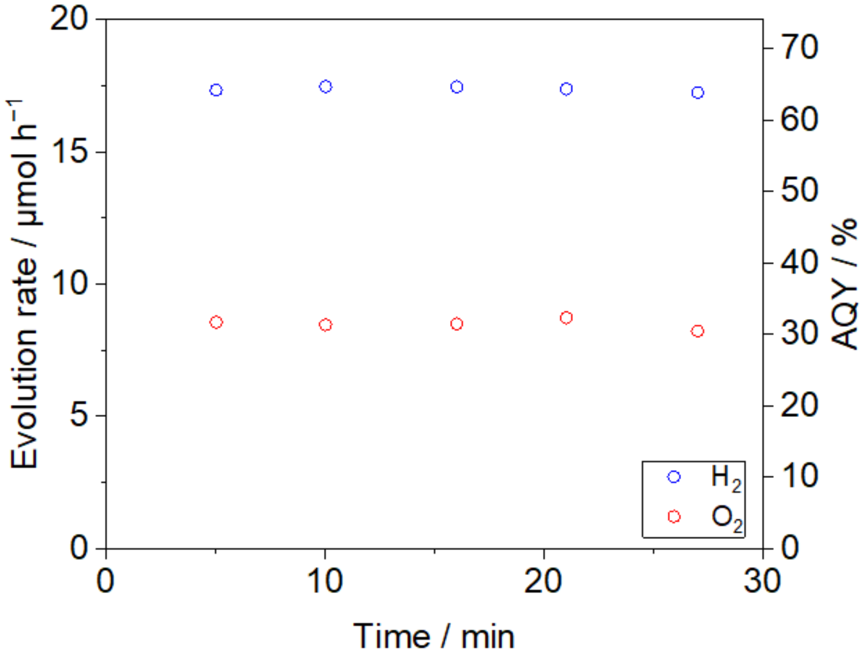

Supplementary Fig. 9: Dependence of H_2 and O_2 evolution rate of TiO_x coated, $CoOOH/Cr_2O_3/Rh$ loaded $SrTiO_3$:Al. These demonstrations were under saturated water vapor balanced with Ar at 24 °C (10 mL min^{-1}, p_{H2O} = 2.9 kPa). Light source: 370 nm LED (5.1 mW cm^{-2}).

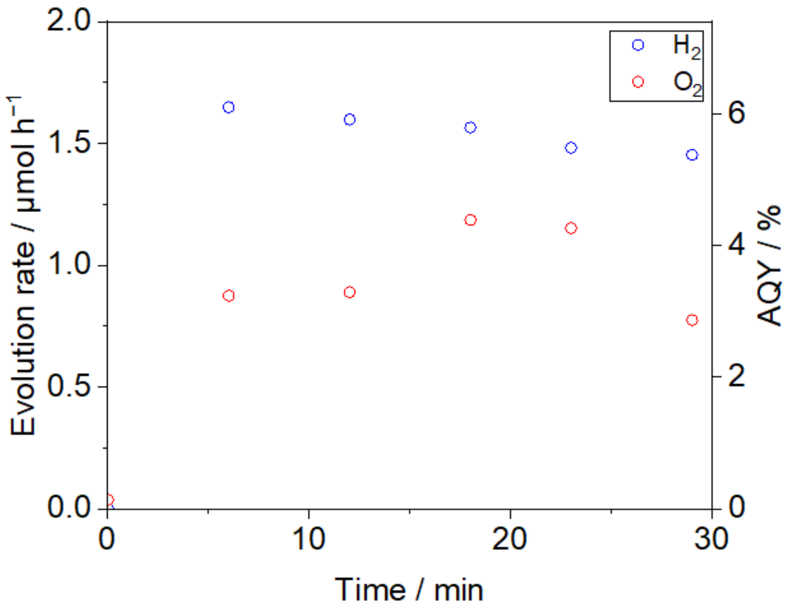

Supplementary Fig. 10: Dependence of H_2 and O_2 evolution rate of physical mixture of CoOOH/Rh loaded $SrTiO_3$:Al and TiO_2 (anatase, 10 wt%). These demonstrations were under saturated water vapor balanced with Ar at 24 °C (10 mL min^{-1}, p_{H2O} = 2.9 kPa). Light source: 370 nm LED (5.1 mW cm^{-2}).

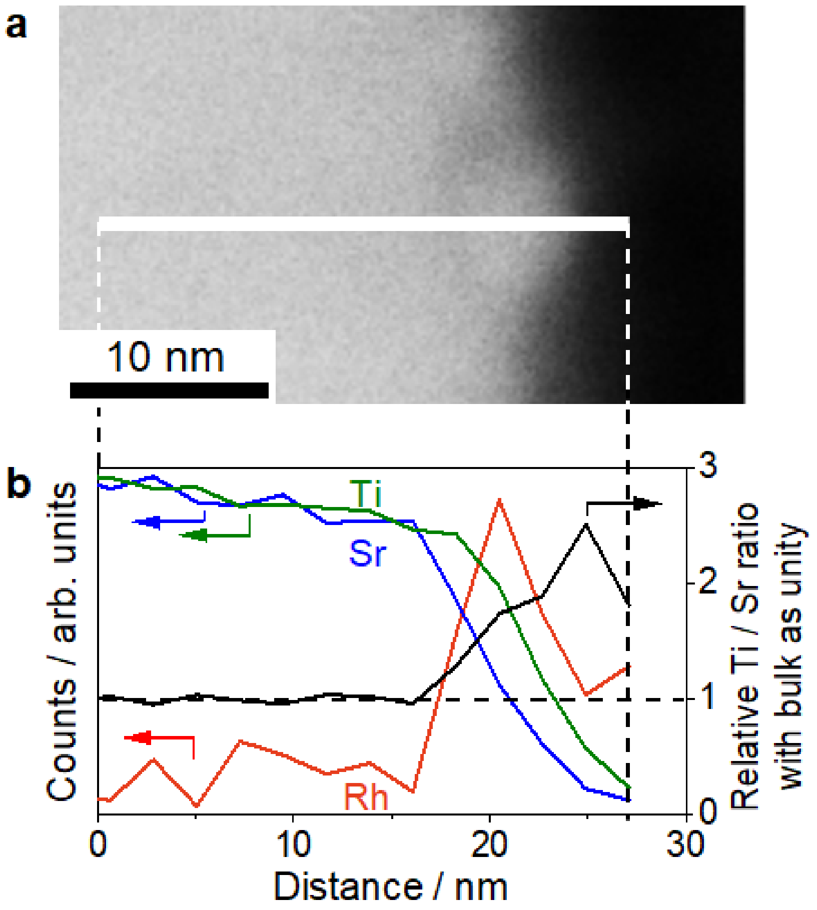

Supplementary Fig. 11: EDS line profile of the Rh nanoparticle with a Ti-rich surface of TiO_x coated, CoOOH/Rh loaded $SrTiO_3$:Al. a Dark field STEM image. **b** EDS line profile.

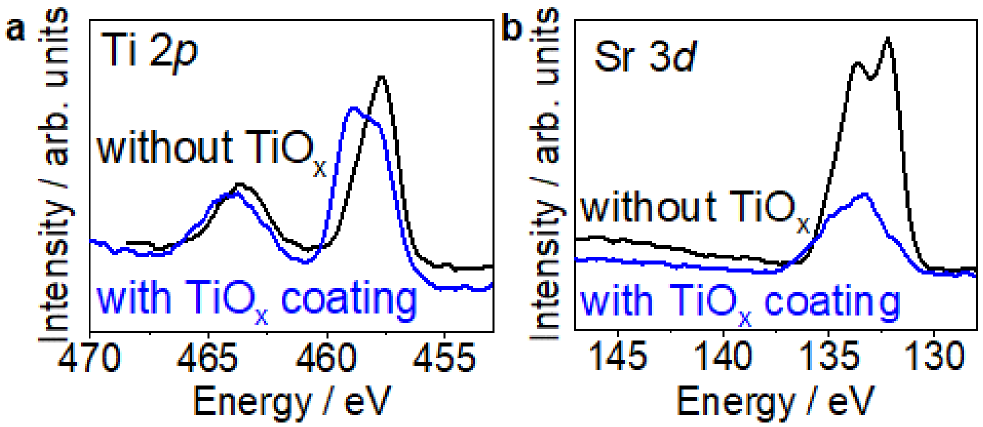

c

Photocatalyst	Ti / Sr ratio
CoOOH/Rh loaded $SrTiO_3$:Al	0.93
TiO_x coated, CoOOH/Rh loaded $SrTiO_3$:Al	5.4

Supplementary Fig. 12: XPS spectra of CoOOH/Rh loaded $SrTiO_3$:Al or TiO_x coated, CoOOH/Rh loaded $SrTiO_3$:Al. **a** Ti 2*p* region spectra. **b** Sr 3*d* region spectra. **c** Table of the atomic ratio of Ti and Sr from the peak area.

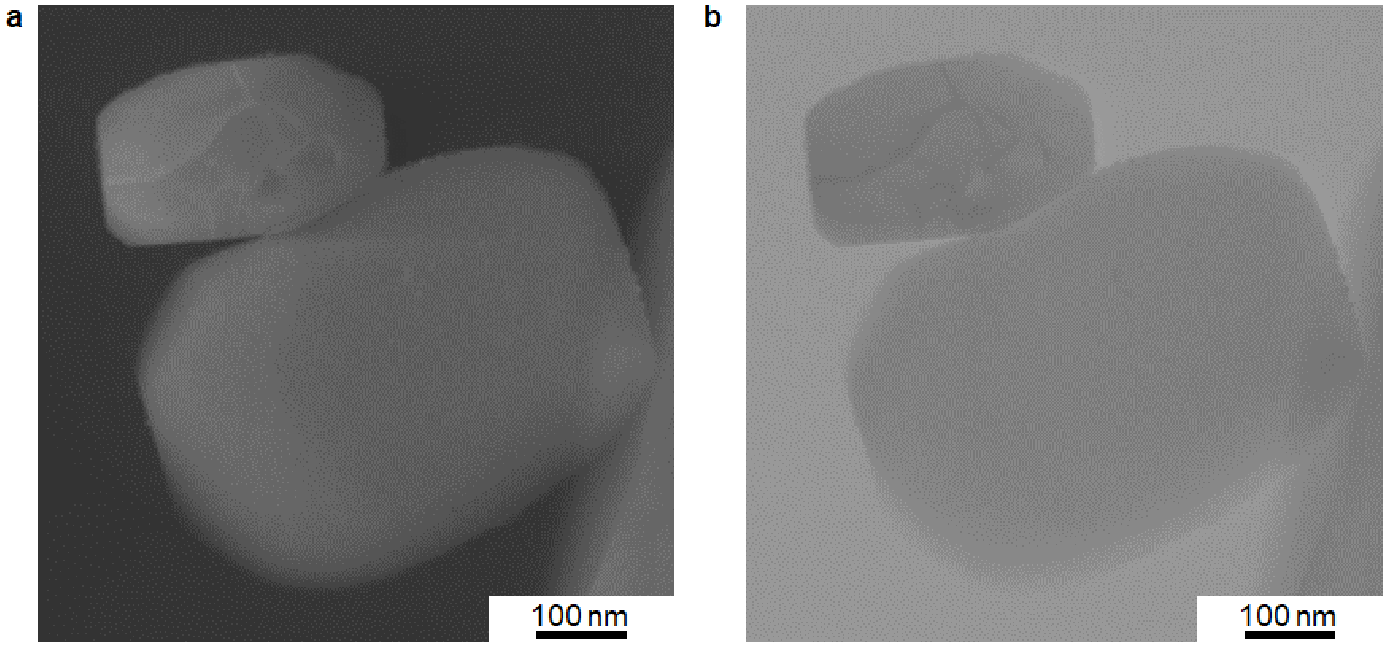

Supplementary Fig. 13: STEM images of TiO_x coated, CoOOH/Rh loaded $SrTiO_3$:Al. a Dark field STEM image. **b** Bright field STEM image.

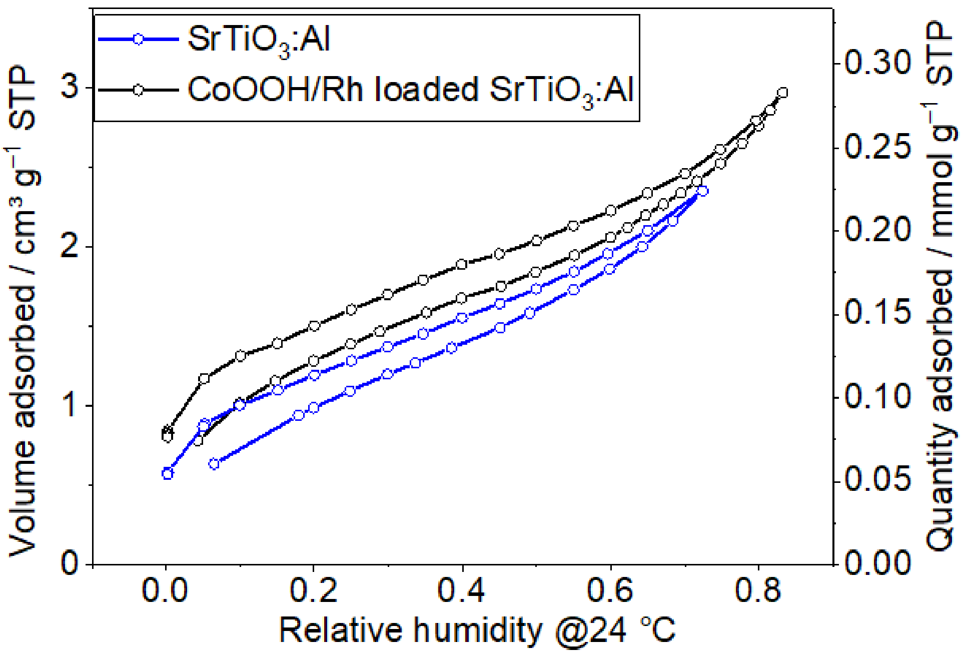

Supplementary Fig. 14: Water adsorption isotherm of $SrTiO_3$:Al. The comparison of water adsorption isotherms between pristine $SrTiO_3$:Al (without any cocatalyst) and $SrTiO_3$:Al with CoOOH and Rh.

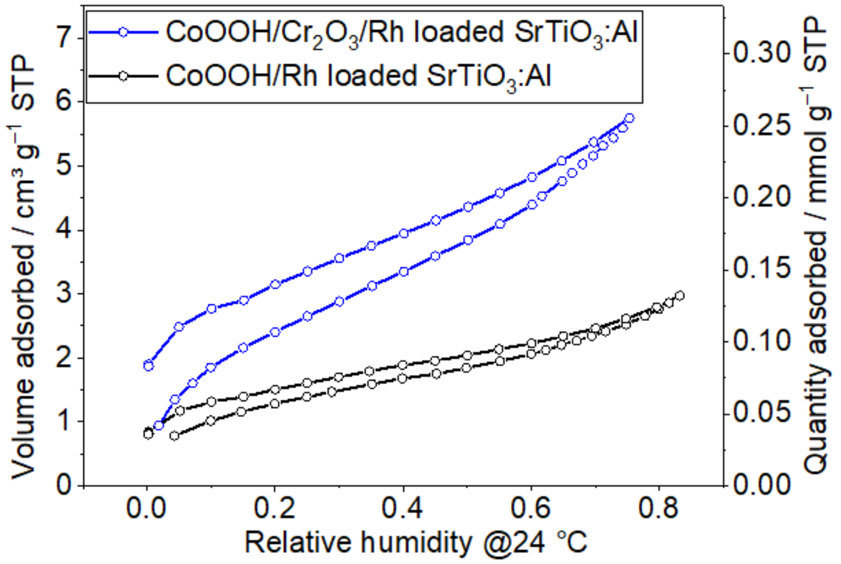

Supplementary Fig. 15: Water adsorption isotherm of CoOOH/Cr_2O_3/Rh loaded $SrTiO_3$:Al. The comparison of water adsorption isotherms between $SrTiO_3$:Al with CoOOH and Cr_2O_3/Rh, and $SrTiO_3$:Al with CoOOH and Rh.

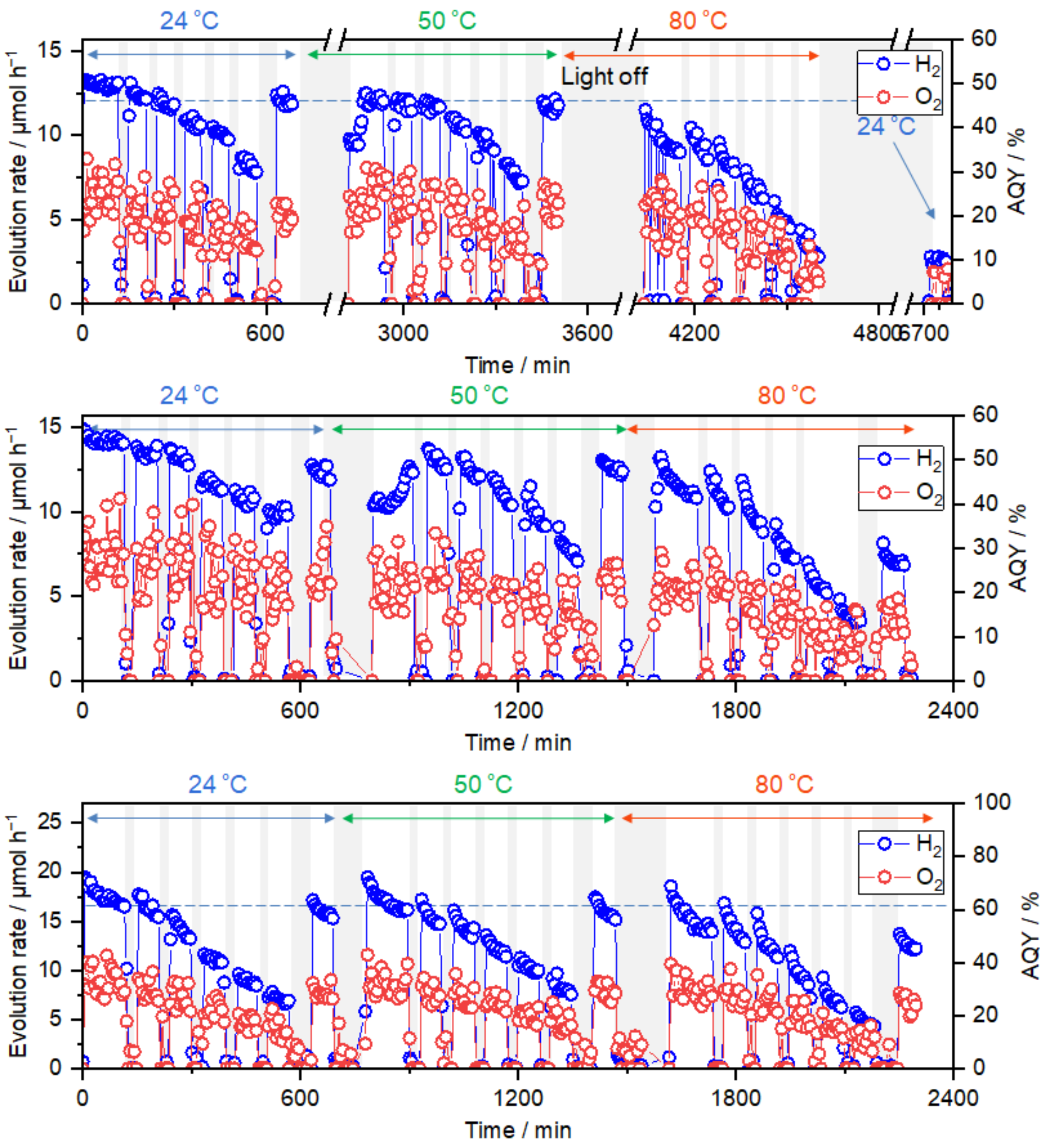

Supplementary Fig. 16: H_2 evolution rate of CoOOH/Rh loaded $SrTiO_3$:Al coated with TiO_x as a function of relative humidity at 24, 50, and 80 °C. Relative humidity was changed to 1, 0.8, 0.6, 0.4, 0.2, 0.1, and 1 every time after turning off the light at each temperature. The measurements were conducted three times with the catalysts prepared at different times.

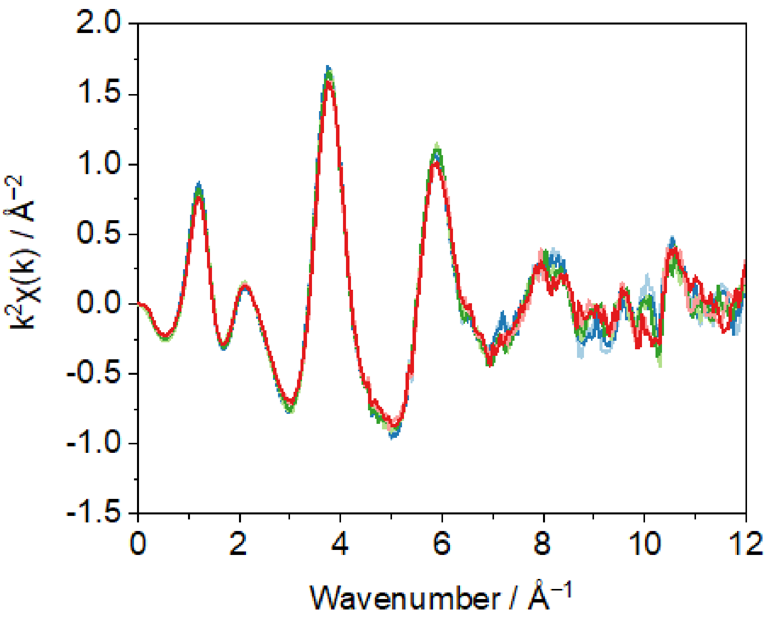

Supplementary Fig. 17: EXFS spectra. EXAFS region of Ta L_{III} XAFS spectra of TaO_x coated, CoOOH/Rh loaded $SrTiO_3$:Al as made (blue) after heat treatment at 60 °C (dry: green, under vapor feeding: light green) and after heat treatment at 250 °C (dry: red, under vapor feeding: pink).

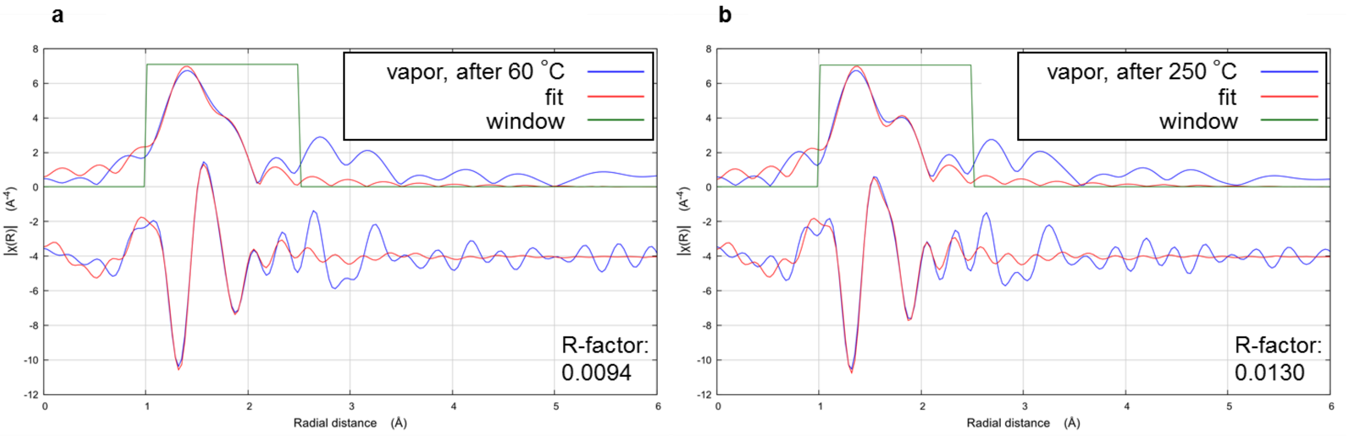

Supplementary Fig. 18: FEFF fitting of EXAFS spectra. FEFF fitting of Ta L_{III} edge EXAFS patterns of CoOOH/Rh loaded $SrTiO_3$:Al coated with TaO_x recorded at 24 °C under saturated water vapor feeding after heat treatment after heat treatment at 60 °C (a) and 250 °C (b). The top lines are Fourier transformed patterns. The bottom lines are the patterns after the back-Fourier transformed patterns of the top lines. Experimental results (blue) were fitted by FEFF code (red). The radial distance range for fittings was 1–2.5 (green).

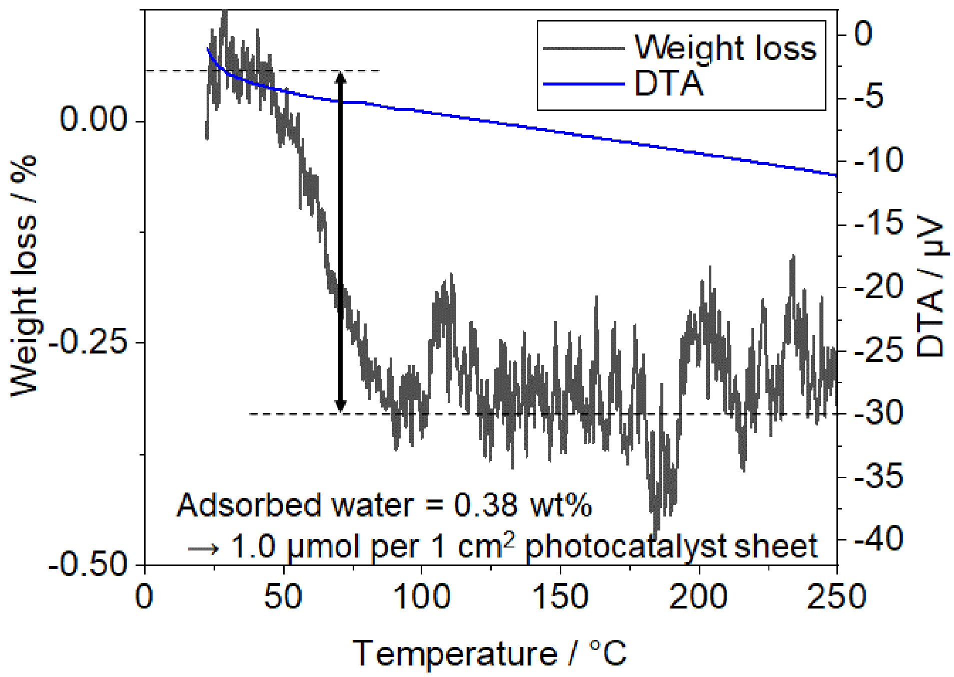

Supplementary Fig. 19: TG-DTA result. TG-DTA curves of TaO_x coated and CoOOH/Rh loaded $SrTiO_3$:Al.

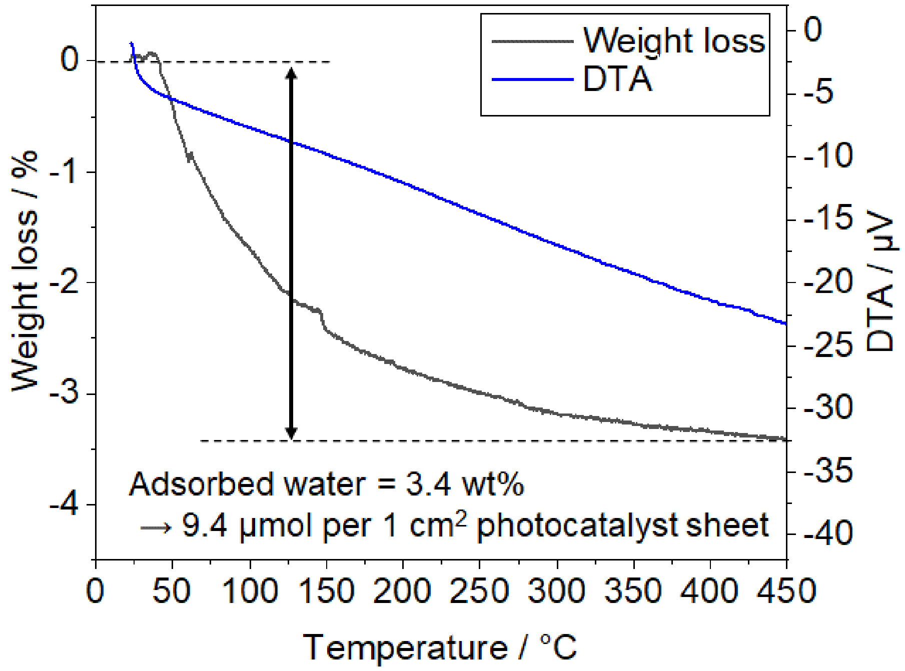

Supplementary Fig. 20: TG-DTA result. TG-DTA curves of TiO_x coated and CoOOH/Rh loaded $SrTiO_3$:Al.

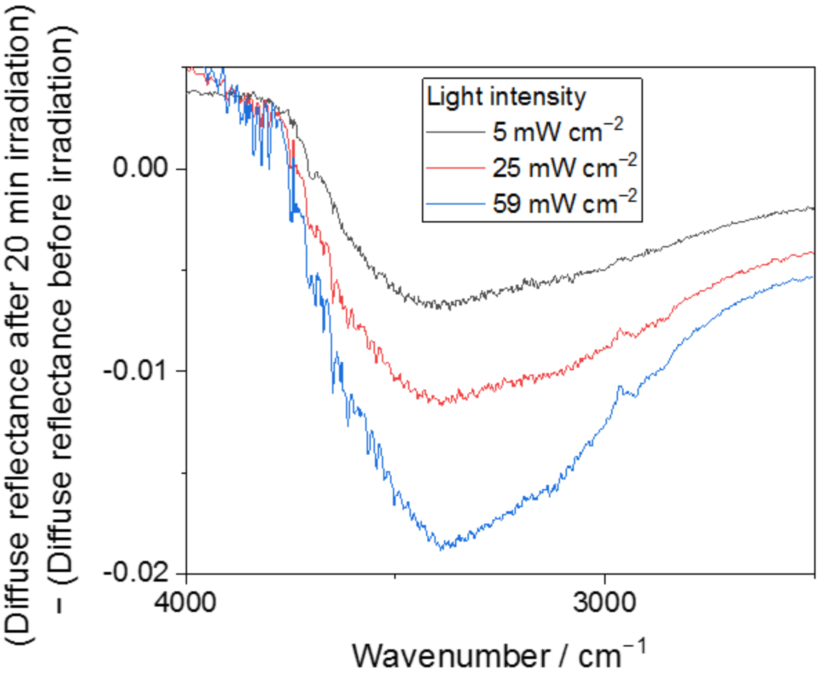

Supplementary Fig. 21: Difference spectra of diffuse reflectance of TiO_x coated, CoOOH/Rh loaded $SrTiO_3$:Al in OH vibration before and after 20 min light irradiation. The feed gas was Ar with saturated water vapor at 24 °C. Incident light: 370 nm LED.
